# Three-dimensional evaluation of mandibular anterior dental crowding in digital dental casts

**DOI:** 10.1590/2177-6709.22.3.064-071.oar

**Published:** 2017

**Authors:** Luciana Quintanilha Pires Fernandes, Livia Kelly Ferraz Nunes, Luana Santos Alves, Felipe de Assis Carvalho Ribeiro, Jonas Capelli

**Affiliations:** 1 Universidade do Estado do Rio de Janeiro, Department of Orthodontics, Rio de Janeiro, RJ, Brazil.

**Keywords:** Orthodontics, Diagnosis, Dental models, Malocclusion

## Abstract

**Introduction:**

Digital dental models provide a more accurate and comprehensive assessment of orthodontic cases. Although this technique is quite promising, there are few three-dimensional measurements methods described in the literature.

**Objective:**

The aim of this study was to propose a method for assessing the degree of mandibular anterior dental crowding in the three planes of space, using digital dental models.

**Methods:**

Thirty dental casts were selected and scanned by Maestro 3D Dental Scanner and imported by Geomagic Qualify 2013 software. The degree of crowding was calculated by two examiners, based on the Little’s Irregularity Index, by means of the definition of axial, coronal and sagittal planes for each model. Intraexaminer analysis was performed with Dahlberg’s Formula (DF) and Intraclass Correlation Coefficients (ICC), and interexaminer analysis was performed with ICC and paired *t*-test.

**Results:**

The ICC showed an excellent agreement (*p* < 0.05) for all measurements, except for the intraexaminer and interexaminer in the Z-axis, in which it was found a moderate agreement. The DF showed a satisfactory accuracy with all measurements showing less than 1 mm difference. The paired *t*-test showed statistical difference between the examiners in two measurements, although it was clinical irrelevant.

**Conclusion:**

When comparing the three planes of space, the Z-axis showed the greatest variation in landmarks placement; however, overall, the present method seems precise and reproducible.

## RESUMO

**Introdução:**

os modelos digitais fornecem uma avaliação mais precisa e abrangente dos casos ortodônticos. Embora essa técnica seja bastante promissora, existem poucos métodos de medição tridimensional descritos na literatura.

**Objetivo:**

o objetivo do presente estudo foi propor um método para avaliar o grau de apinhamento dentário anteroinferior, nos três planos do espaço, usando modelos digitais.

**Métodos:**

trinta modelos de gesso foram selecionados, escaneados pelo *scanner* Maestro Dental 3D e importados para o *software* Geomagic Qualify 2013. Dois examinadores calcularam o grau de apinhamento, com base no Índice de Irregularidade de Little, mediante a definição dos planos axial, coronal e sagital para cada modelo. A análise intraexaminador foi realizada por meio da fórmula de Dahlberg (FD) e do Coeficiente de Correlação Intraclasse (ICC), enquanto a análise interexaminadores foi realizada com o ICC e o teste *t* pareado.

**Resultados:**

o ICC mostrou um excelente grau de concordância (*p*< 0,05) para todas as medições, exceto para as avaliações intraexaminador e interexaminadores no eixo Z, onde foi encontrado um grau moderado. A FD mostrou uma precisão satisfatória, com todas as medidas apresentando diferença menor que 1 mm. O teste *t* pareado mostrou diferenças estatisticamente significativas entre os examinadores em duas medidas, apesar de terem sido clinicamente irrelevantes.

**Conclusão:**

ao se comparar os três planos do espaço, o eixo Z mostrou maior variação na marcação de pontos; no entanto, em geral, o presente método parece ser preciso e reprodutível.

## INTRODUCTION

Dental casts are widely used in dental practice as well as on research projects. In Orthodontics, it is considered to be of extreme value for diagnosis and the clinical decision making process. With the introduction of three-dimensional scanners, it has become possible to obtain digital dental models aiming the study of dental arches. These models feature precision and can be manipulated through specific software, enabling the completion of its analysis, which is an important step for orthodontic treatment planning.^1^

Among the many advantages attributed to the digital dental models, it can be cited the easy manipulation and the fact that they do not require physical space to be stored. They are saved on the computer, producing durable images without loss or damage to the original models.^2^ ^-^ ^4^Certain limitations of plaster dental casts, as its manufacturing time and its weight and volume, would be eliminated with the use of digital dental models, especially with the increased demand for information exchange and ease of communication between professionals.^5^ ^-^ ^7^

Digital dental models acquisition by computed tomography,^8^ optical scanning of plaster dental casts,^3^ ^,^ ^4^ ^,^ ^7^ ^,^ ^9^ ^-^ ^13^ digital dental models obtained by directly scanning the alginate impressions^14^ and through two-dimensional photographs^2^ have been evaluated and compared to conventional plaster dental casts. According to these studies, all of these methods were reliable and accurate.

Some studies have analyzed the reliability of the digital dental models compared to conventional exams, by comparing linear measurements in both digital and plaster dental casts.^2^ ^-^ ^5^ ^,^ ^7^ ^-^ ^16^ Among the parameters chosen to assess the reproducibility and reliability of digital dental models, the most used were the dental discrepancy,^8^ ^,^ ^13^ ^,^ ^14^ the mesiodistal tooth dimension,^3^ ^,^ ^4^ ^,^ ^7^ ^,^ ^10^ ^,^ ^11^ arch width measurements,^4^ ^,^ ^9^ ^,^ ^10^ ^,^ ^12^ overbite and overjet^3^ ^,^ ^9^ ^-^ ^11^ and midline discrepancy.^3^ ^,^ ^9^ ^,^ ^10^ The virtual orthodontic planning (setup) also became possible with the introduction of digital dental models.^17^ ^,^ ^18^ Im et al^19^ found intra-arch and interarch similar measurements when comparing virtual and manual tooth setups.

Although an extensive literature proving digital dental models can substitute plaster dental casts with satisfactory degrees of accuracy and reproducibility of linear measurements,^20^ only few studies used methodologies that evaluated the parameters through the Cartesian coordinate system. Described by Renee Descartes (1596-1650), the coordinate system allows to determine the specific localization of each point in the three dimensions of space, by projecting its localization in the three fixed perpendicular planes.^21^

Mandibular anterior crowding often leads patients to seek for dental alignment; being so, this malocclusion should be investigated, in order to obtain an appropriate treatment plan. Many methods have been proposed to evaluate this malocclusion^22^ ^-^ ^29^ and the most used is the Irregularity Index described by Little in 1975.^30^ This method aims to quantify the degree of crowding in the mandibular anterior region by measuring the linear displacement of the anatomic contact points of each incisor from the adjacent tooth anatomic point, assessing the degree of tooth misalignment in the horizontal plane.

The goal of this study was to propose a method to assess the degree of mandibular anterior dental crowding in the three planes of space, using Cartesian coordinates on digital dental models.

## MATERIAL AND METHODS

After approval by the Institution Ethical Committee, thirty lower plaster dental casts were selected from the diagnostic records of patients that were receiving orthodontic treatment at the Orthodontic Clinic of the *Universidade do Estado do Rio de Janeiro* (UERJ, Brazil).

The inclusion criteria were lower dental casts with complete permanent dentition, with or without the second and third molars. The first molars should have their five cusps relatively equidistant in relation to the occlusal plane. Models with tipped first molars and/or lacking structural integrity could compromise the measurements and thus were excluded from this study.

The dental casts were digitized by using the Maestro 3D Dental Scanner, MDS300 Model (AGE Solutions, Potedera, Italy), which uses the structured light for its image acquisition method. The images were automatically processed by the Maestro Easy Dental Scan software (4.018.086.4295), generating files with the .STL (stereolithography) extension for each dental model. Three-dimensional images were analyzed by Geomagic Qualify 2013 software (Raindrop GeomagicInc, Cary, NC).

An individual coordinate system was created for each digital dental model in order to permit a customized assessment of them in the three planes of space, as proposed in this study. The whole process was made by two examiners, who were previously calibrated before performing the landmark placement and measurements.

### Axial, coronal and sagittal planes

**Figure 1:**
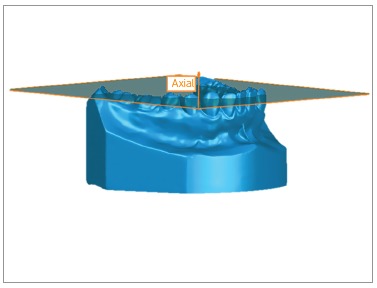
Axial plane.

It was established that the axial plane should be coincident with the patients’ occlusal plane. Three points defined it: mesiobuccal cusp tip of both lower first molars and the contact point between lower central incisors. If the patient had a diastema in this region, the third point was marked where it should be the contact point in the lower right central incisor. This plane was related to the X and Y-axes (Fig 1).

**Figure 2:**
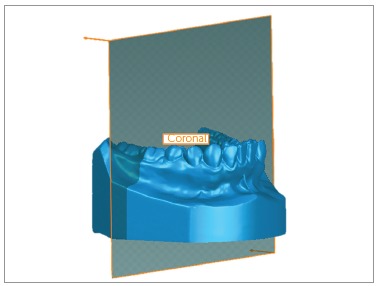
Coronal plane.

Three points also defined the coronal plane: mass center (centroid) of the dental crowns of both lower first molars and center of the mesiodistal groove of the right lower first molar. This plan was related to the X and Z-axes (Fig 2).

**Figure 3:**
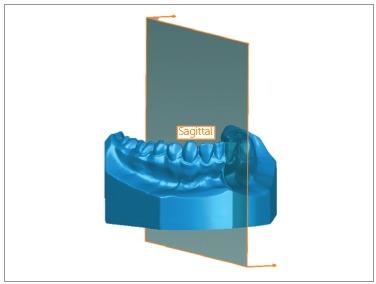
Sagittal plane.

Sagittal plane was defined as being perpendicular to the two plans previously described, passing through the contact point between lower central incisors. If the patient had a diastema in this region, the third point was marked where it should be the contact point in the lower right central incisor. This plan was related to the Y and Z-axes (Fig 3).

### Coordinate system definition

**Figure 4:**
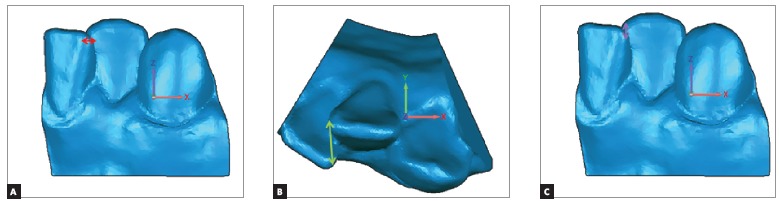
A) Transversal displacement between left central lower incisor and left lateral lower incisor (X-axis). B) Anterior-posterior displacement between left central lower incisor and left lateral lower incisor (Y-axis). C) Vertical displacement between left central lower incisor and left lateral lower incisor (Z-axis).

For the custom-made coordinate system described above, changes in the X-axis reflected transversal displacements in the anterior region of the digital dental models (Fig 4A), while changes in the Y-axis represented anterior-posterior changes (Fig 4B). Differences in the Z-axis meant vertical changes (Fig 4C).

**Figure 5:**
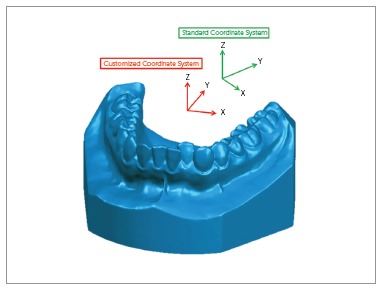
Coordinate system: the red axes correspond to the customized coordinate system created for this model; the green axes correspond to the standard coordinate system that the software presents.

Usually, while creating a digital dental model, it is placed in a standard coordinate system according to the spatial position of the dental model in the scanner at the time of acquisition. Aiming proper measurements as planned, it was mandatory to set the software to use the newly defined coordinate system instead of the standard one. From this time-point on, all markings and measurements made were related to the customized coordinate system (Fig 5).

### Landmark placement

**Figure 6:**
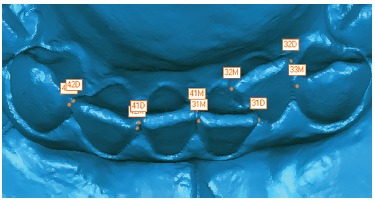
Points used to evaluate the mandibular anterior dental crowding.

In order to quantify the degree of crowding in the mandibular anterior region of each dental model, it was necessary to mark the contact points of the incisors and its adjacent teeth. Thus, the anatomic points from the mesial of the right canine to the mesial of the left canine were marked, as proposed by the Irregularity Index.^30^ The digital images of the dental models could be rotated around any axis and enlarged on screen to facilitate the landmark placement. The objective was to achieve high accuracy in defining the exact location of these points (Fig 6).

### Degree of crowding measurement in the three planes of space

**Figure 7:**
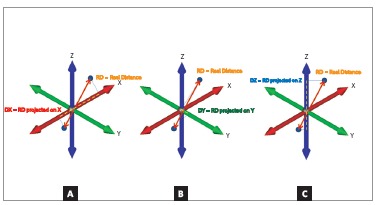
Graphic representation of the projections of the RD on the: A) X-axis (DX); B) Y-axis (DY); C) Z-axis (DZ).

The next step was to measure the linear displacement of the contact points in this region. As proposed by this study, it was evaluated not only the real distance (RD), defined as the linear distance between two points without projecting its localization in the three planes of space (the distance obtained when a dental cast is measured with a digital caliper, for example), but the measurements of the projections of the RD on the three axes (X, Y and Z), separately (Fig 7). So, it was obtained four measurements for each pair of points (RD, DX, DY and DZ), which allow to decompose the RD identifying how much anterior-posterior (DY), transversal (DX) and vertical (DZ) components were present.

All data was recorded and compiled in a spreadsheet, followed by the statistical analysis. All of the values found for the RD, DX, DY and DZ were added, in an analogous way to that described by Little,^30^ to obtain the final crowding value of the assessed region. Thus, the DX found between the five pairs of points (mesial of right canine to the distal of right lateral incisor, mesial of right lateral incisor to the distal of right central incisor, mesial of right central incisor to the mesial of left central incisor, distal of left central incisor to the mesial of left lateral incisor and distal of left lateral incisor to the mesial of left canine) were added and the anterior crowding in the transversal axis were obtained. Similarly, the anterior crowding in the anterior-posterior axis (DY) and in the vertical axis (DZ) were calculated, as well as the total crowding calculated based on the RDs.

### Statistical analysis

Statistical analysis was done with the IBM SPSS Statistics 22 software. The sample size calculation was carried out using an expected difference of 0.5mm, with 90% power and an 5% alpha level. The Shapiro-Wilk test found normal distribution and the Levene test verified the equality of variances. Two examiners made the whole process of landmark placement and measurements, in order to assess the reproducibility and the interexaminer precision of the proposed method, which was performed with Intraclass Correlation Coefficient (ICC) and with paired *t*-test. Ten randomly selected digital dental models had all measurements repeated after fifteen days by both examiners, in order to evaluate the reproducibility and the intraexaminer precision, which were evaluated with Intraclass Correlation Coefficient (ICC) and with Dahlberg’s Formula (DF).

## RESULTS

The Shapiro-Wilk test indicated a normal distribution for the sample, and the sample’s descriptive analysis is shown in Table 1.

**Table 1:** Descriptive analysis: DX, real distance projected on the X axis; DY, real distance projected on the Y axis; DZ, real distance projected on the Z axis; RD, real distance; E1, examiner 1; E2, examiner 2.

	DX		DY		DZ		RD	
	E1	E2	E1	E2	E1	E2	E1	E2
Sample size	30	30	30	30	30	30	30	30
Minimum (mm)	1	1.3	0.5	0.8	1	0.8	2.5	2.9
Maximum (mm)	5.3	5.8	8.7	8.8	4.8	8.3	11.7	13.2
Mean (mm)	2.6	3.1	3.8	3.8	2.5	2.8	5.8	6.3
Standard deviation (mm)	1.1	1.2	2.2	2.2	1	1.5	2.4	2.5

The DF showed a satisfactory precision of all measurements (DX, DY, DZ and RD) showing less than 1 mm difference (Table 2). The less precise measurement found by examiner 1 (E1) was related to the Z-axis (DZ), while for examiner 2 (E2) was related to the real distance (RD). The most precise measurement found by E1 was related to the X-axis (DX), while for E2 was related to Y-axis (DY).

**Table 2:** Dahlberg’s Formula (DF): DX, real distance projected on the X axis; DY, real distance projected on the Y axis; DZ, real distance projected on the Z axis; RD, real distance; E1, examiner 1; E2, examiner 2.

	DX (mm)	DY (mm)	DZ (mm)	RD (mm)
Intraexaminer (E1) DF	0.19	0.44	0.58	0.46
Intraexaminer (E2) DF	0.62	0.47	0.64	0.8

The ICC showed an excellent agreement for almost all measurements, except for intraexaminer of examiner 1 in the Z-axis and interexaminer in the Z-axis, where it observed a moderate agreement (Table 3). The paired *t*-test showed statistical differences between both examiners for real distance (RD) and for the X-axis (DX). The results were considered significant at *p*< 0.05 (Table 4).

**Table 3:** Intraexaminer and Interexaminer Intraclass Correlation Coefficient (ICC) : DX, real distance projected on the X axis; DY, real distance projected on the Y axis; DZ, real distance projected on the Z axis; RD, real distance; E1, examiner 1; E2, examiner 2.

	DX	DY	DZ	RD
Intraexaminer (E1) ICC	0.97	0.92	0.7	0.96
	p < 0.0001	p < 0.0001	p = 0.0063	p < 0.0001
Intraexaminer (E2) ICC	0.8	0.98	0.82	0.92
	p = 0.0014	p < 0.0001	p = 0.0009	p < 0.0001
Interexaminer ICC	0.76	0.85	0.65	0.79
	p < 0.0001	p < 0.0001	p < 0.0001	p < 0.0001

**Table 4:** Paired *t*-test: DX, real distance projected on the X axis; DY, real distance projected on the Y axis; DZ, real distance projected on the Z axis; RD, real distance; E1, examiner 1; E2, examiner 2; SD, standard deviation.

	DX		DY		DZ		RD	
	E1	E2	E1	E2	E1	E2	E1	E2
Mean	2.57	3.08	3.85	3.82	2.55	2.82	5.8	6.35
SD	1.11	1.21	2.19	2.16	0.98	1.55	2.37	2.5
Difference between examiners	0.51		0.03		0.27		0.55	
p value	< 0.0001		0.8979		0.1600		0.0090	

## DISCUSSION

The present study’s choice for evaluation of the mandibular anterior crowding is justified by the clinical relevance of this malocclusion. To assess the degree of mandibular anterior crowding, it was chosen the Irregularity Index as the measurement parameter, since this is the most accepted and used method by orthodontists. However, since this index was described without the support of digital images, it has limitations, as the tooth misalignment is seen only in the horizontal plane.^30^ The method proposed by this study allows a more comprehensive assessment of crowding, quantifying the degree of this malocclusion not only in the horizontal plane but also in the vertical and transverse planes. This way, when evaluating crowding using this three-dimensional method, the orthodontist can distinguish the degree of misalignment and unleveling in the mandibular anterior teeth, separately.

When proposing a new method, both precision and accuracy should be evaluated. However, to measure accuracy, it would be necessary to compare the measures obtained from the digital models to the supposed measures obtained from the plaster models, since this is the gold standard up to now. Nevertheless, it is impossible to measure the distance between two points, projected on the three planes of space, in plaster dental casts. So, this study aimed to measure only precision, and not accuracy. To measure interexaminer precision, thirty different dental casts were measured, based on the sample calculation’s result. It is expected to observe higher differences when evaluating interexaminer than intraexaminer precision. So, to evaluate the intraexaminer precision, it would be necessary less repeated measurements than to evaluate the interexaminer precision. Since it was evaluated thirty dental casts by both examiners in order to evaluate the interexaminer precision, we decided to measure only ten dental casts in order to evaluate intraexaminer precision.

We believe that the coordinate system created by this study is user friendly, since the chosen points were considered to be easy to identify and mark on digital dental models. A possible limitation of this system is the reference of the sagittal and axial planes between the central incisors, since the evaluated region has a parabolic shape that will create an angle in relation to the sagittal plane when evaluating the canines’ position. Nevertheless, since this method was proposed only to the anterior teeth, the degree of distortion embedded in the obtained measurements is irrelevant. Another limitation of this method is the molar used as reference for the occlusal plane definition; so, this method is applicable only when the first molars are in a proper position. In cases with tipped first molars or without them, we suggest the use of premolars as a substitute reference for the occlusal plane definition, although this new coordinate system should be evaluated by other studies.

The greatest observed difference between the compared groups was of 0.8 mm, which grants this method a high precision. When comparing the results found for the three axes separately, it was observed that the Z-axis showed the worst result with bigger differences in both intraexaminer evaluations. So, based on the results of this study, when evaluating mandibular anterior crowding as proposed by this work, the operator should carefully mark the points, especially in the vertical direction. The authors believe that this difference might have occurred due to the fact that the cervical-occlusal distance of the anterior teeth is bigger than the buccal-lingual distance; so, it is expected to observe more landmark variations in the vertical direction, when comparing to the anterior-posterior and transverse directions.

The paired *t*-test showed a statistical difference between both examiners when comparing RD and DX measurements, although it was an irrelevant clinical difference (0.55 mm and 0.51 mm, respectively; hence, 0.05 mm and 0.01 mm higher than the value stipulated in the sample size calculation). The greatest difference observed in the RD is because it represents the projections of the axes (X, Y, Z), so small differences in each axis are summed in the RD.

Although the digital dental models are well known and recently widely studied, it can still be considered a new diagnostic approach, not being used very often by most orthodontists worldwide, since there are additional costs that could limit the accessibility and the diffusion of this three-dimensional method. Moreover, as any other technique, there is a learning curve for the clinician to be proficient in the use of digital dental models.

The goal of this study was to propose a diagnostic tool that allows a comprehensive assessment of the mandibular anterior crowding through the three-dimensional analysis that is available on the Geomagic Qualify software. More studies should be carried out using a true three-dimensional analysis of digital dental models, through a customized coordinated system, in order to corroborate and/or reject the findings of the present study aiming to supply clinicians the best and accurate tools possible to support diagnosis, treatment planning at the same time it provides a more effective assessment of treatment results.

## CONCLUSIONS

The results of this study indicated that:

» The proposed method of assessing the degree of mandibular anterior dental crowding in the three planes of space, using Cartesian coordinates in digital dental models, is precise and reproducible, although the accuracy of the method was not tested.

» When comparing the three planes separately, it was concluded that the Z-axis showed the greatest variation in landmarks placement, indicating that it is necessary a special attention in vertical direction when evaluating mandibular anterior dental crowding using the method proposed by this study.
